# MicroRNAs-Proteomic Networks Characterizing Human Medulloblastoma-SLCs

**DOI:** 10.1155/2016/2683042

**Published:** 2016-01-06

**Authors:** Giuseppina Catanzaro, Zein Mersini Besharat, Neha Garg, Maurizio Ronci, Luisa Pieroni, Evelina Miele, Angela Mastronuzzi, Andrea Carai, Vincenzo Alfano, Agnese Po, Isabella Screpanti, Franco Locatelli, Andrea Urbani, Elisabetta Ferretti

**Affiliations:** ^1^Department of Molecular Medicine and Experimental Medicine, Sapienza University, 00161 Rome, Italy; ^2^Department of Molecular Medicine, Sapienza University, 00161 Rome, Italy; ^3^Santa Lucia IRCCS Foundation, 00143 Rome, Italy; ^4^Department of Medical, Oral and Biotechnological Sciences, University G. d'Annunzio of Chieti-Pescara, 66013 Chieti, Italy; ^5^Mawson Institute, University of South Australia, Mawson Lakes, SA 5095, Australia; ^6^Department of Experimental Medicine and Surgery, University of Rome “Tor Vergata”, 00133 Rome, Italy; ^7^Center for Life NanoScience at Sapienza, Italian Institute of Technology, Viale Regina Elena 291, 00161 Rome, Italy; ^8^Department of Hematology/Oncology and Stem Cell Transplantation, Bambino Gesù Children's Hospital, IRCCS, 00165 Rome, Italy; ^9^Department of Neuroscience and Neurorehabilitation, Neurosurgery Unit, Bambino Gesù Children's Hospital, IRCCS, 00165 Rome, Italy; ^10^Department of Pediatric Science, University of Pavia, 27100 Pavia, Italy

## Abstract

Medulloblastoma (MB) is the most common malignant brain tumor of pediatric age and is characterized by cells expressing stem, astroglial, and neuronal markers. Among them, stem-like cells (hMB-SLCs) represent a fraction of the tumor cell population with the potential of self-renewal and proliferation and have been associated with tumor poor prognosis. In this context, microRNAs have been described as playing a pivotal role in stem cells differentiation. In our paper, we analyze microRNAs profile and genes expression of hMB-SLCs before and after Retinoic Acid- (RA-) induced differentiation. We aimed to identify pivotal players of specific pathways sustaining stemness and/or tumor development and progression and integrate the results of our recent proteomic study. Our results uncovered 22 differentially expressed microRNAs that were used as input together with deregulated genes and proteins in the Genomatix Pathway System (GePS) analysis revealing 3 subnetworks that could be interestingly involved in the maintenance of hMB-SLCs proliferation. Taken together, our findings highlight microRNAs, genes, and proteins that are significantly modulated in hMB-SLCs with respect to their RA-differentiated counterparts and could open new perspectives for prognostic and therapeutic intervention on MB.

## 1. Introduction

Aggressive multimodal therapy has significantly improved medulloblastoma (MB) outcomes but up to 30% of the cases still recur and treated patients got debilitating secondary sequelae [1]. MB is characterized by significant intratumoral heterogeneity and comprised of cells expressing stem, astroglial, and neuronal markers whose contribution to tumor expansion has not been completely understood yet [2]. Our and other laboratories have provided evidence that MB harbors a distinct subpopulation of stem cells or cancer stem-like cells (SLCs) [3, 4] identified by the marker expression of Nanog [3]. Importantly stem cell signatures have been associated with tumor poor prognosis and, very recently, we characterized SLCs in MB with aggressive behavior [1]. Interestingly, it has been reported that clonal genetic events observed in metastases can be demonstrated in a restricted subclone of the primary tumor, suggesting that only rare cells have the ability to metastasize [5]. SLCs have been proposed as the major source of resistance toward conventional therapy [6] and a never-ending reservoir for cancer maintenance and progression [7]. Knowledge of the SLCs molecular features is urgently needed to understand tumor progression and to design novel stem specific therapeutic strategies. About this topic, we previously isolated SLCs from human MBs (hMB-SLCs) [1, 3] and more recently investigated the proteomic profile of hMB-SLCs and of their RA-differentiated counterparts applying a label-free quantitative proteomic analysis able to maximize the identification capacities of the statistically differential spectral features [8]. In MB microRNAs, noncoding RNAs that control gene expression [9] have been described as being deregulated with respect to normal cerebellum [10], form regulatory networks with components of signaling pathways deregulated in cancer cells [11], and have also been described to play a pivotal role in stem cell differentiation [12]. In new experiments we further characterized expression of microRNA and genes hMB-SLCs and this paper reports a specific analysis of proteins, microRNAs, and genes that regulate stem cell maintenance. Since the identification of specific pathways supporting the survival of SLCs could open new perspectives in cancer treatment, using the Genomatix Pathway System (GePS) analysis, we also performed a deep network pathway analysis with the aim of building regulatory networks that include the crosstalk among microRNAs, mRNAs, and proteins to better define SLCs specific signaling components.

## 2. Materials and Methods

### 2.1. Materials

Unless otherwise indicated, media and supplements were purchased from Gibco-Invitrogen (Carlsbad, CA) and chemicals were purchased from Sigma-Aldrich (St. Louis, MO).

### 2.2. Culture of hMB-SLCs

Human medulloblastoma samples (MB) were collected during surgical resection with the approval of institutional review board as described earlier [13]. Tissues were collected in Hank's Balanced Salt Solution (HBSS) supplemented with 0.5% glucose and penicillin-streptomycin, grossly triturated with serological pipette, and treated with DNAse I to a final concentration of 0.04% for 20 min. Subsequently, cell aggregates were mechanically disrupted using pipettes of decreasing bore size to obtain a single cell suspension. After dissociation and centrifugation, cells were cultured as oncospheres in selective medium, DMEM/F12 supplemented with 0.6% glucose, 60 mg/mL N-acetyl-L-cysteine, 2 mg/mL heparin, 20 ng/mL NGF, 20 ng/mL bFGF (Peprotech, Rocky Hill, NJ), 1x penicillin-streptomycin, and B27 supplement without vitamin A. For differentiation studies, oncospheres were mechanically dissociated and plated on D-poly-lysine coated dishes in differentiation medium (DMEM/F12 with N2 supplement and 2 mg/mL heparin, 0.6% glucose, 60 mg/mL N-acetyl-L-cysteine containing 1% fetal bovine serum, and RA 8 *μ*M) for 48 h. All samples were prepared in 3 biological replicates for each point.

### 2.3. Immunochemical Analysis

Cells were lysed in Tris-HCl pH 7.6, 50 mM, deoxycholic acid sodium salt 0.5%, NaCl 140 mM, NP40 1%, EDTA 5 mM, NaF 100 mM, Na pyrophosphate 2 mM, and protease inhibitors. Lysates were separated on 10% or 12% acrylamide gel and immunoblotted using standard procedures. Rabbit anti-OCT4, #2750 (Cell Signaling Technology Inc., Danvers, MA), rabbit anti-Nanog, PA1-097 (ThermoFisher Scientific, Rockford, IL), mouse anti-*β*-3-Tubulin (TU-20), #4466 (Cell Signaling Technology Inc.), mouse anti-GFAP, MAB360 (Merck Millipore, Darmstadt), rabbit anti-HspA1A, sc-33575 (Santa Cruz Biotechnology, CA), rabbit anti-PCNA, #13110 (Cell Signaling Technology Inc.), rabbit anti-NPM, #3542 (Cell Signaling Technology Inc.), mouse anti-GAPDH, ab8245 (AbCam, Cambridge, UK), rabbit anti-Hsp60, #D307 (Cell Signaling Technology Inc.), and HRP-conjugated secondary antisera (Santa Cruz Biotechnology, CA) were used followed by enhanced chemiluminescence (ECL Amersham, Amersham, UK) and images were acquired using BioRad ChemiDoc MP Imaging System (BioRad, Hercules, CA). Densitometric analysis was performed using the BioRad associated Image Lab Software (BioRad, Hercules, CA). Values are expressed as fold over internal control, represented by GAPDH or Hsp60, in the case of NPM, whose expressions were not significantly modulated in the proteome profiles.

### 2.4. Bright Field Microscopy

For bright field acquisition, oncospheres were plated on D-poly-lysine coated Lab-Tek chamber slides and allowed to adhere for 3 h. RA-differentiated MB cells (RA) were mechanically dissociated, plated on D-poly-lysine coated Lab-Tek chamber slides, and cultured in differentiating medium for 2 days and then fixed with 4% paraformaldehyde for 10 min at RT. Bright field high-resolution images were acquired using a FV1200 MPE laser scanning confocal microscope (Olympus) with a UPlanSAPO 60x/1.35 NA oil immersion objective.

### 2.5. RNA Isolation and Real-Time qPCR

RNA isolation was performed as described previously [14]. cDNA synthesis was performed using the High Capacity cDNA reverse transcription kit from Applied Biosystems (AB, Foster City, CA). Quantitative reverse transcription (qPCR) analysis of* OCT4*,* Nanog*,* Nestin*,* KLF4*, *β-3-Tubulin*,* GFAP*,* BMP4*,* MSI1*,* BMI1*,* PCNA*,* CCNE1*,* CCNB1*,* CCND1*, and* MID1* mRNA expression was performed on cDNAs employing the Applied Biosystems ViiA 7 Real-Time PCR System using TaqMan gene expression assay according to the manufacturer's instructions (AB). Each amplification reaction was performed in triplicate, and the average of the three threshold cycles was used to calculate the amount of transcripts in the sample (SDS software, AB). mRNA quantification was expressed, in arbitrary units, as the ratio of the sample quantity to the calibrator or to the mean values of control samples. All values were normalized to four endogenous controls, GAPDH, *β*-2 microglobulin, Hprt, and Tbp.

### 2.6. MicroRNA Expression Profiling, Microarray Data Analysis, and qPCR

Analysis of the expression profiling of 754 microRNAs was carried out on 3 replicates for each RNA sample according to Applied Biosystems protocols (Foster City, CA). This array detects the 754 most abundantly expressed and best-characterized microRNAs in the Human microRNA genome. The assay included RT with specific primers followed by real-time qPCR using the TaqMan Array Human microRNA A + B Cards set v3.0 and TaqMan universal master mix in an Applied Biosystems ViiA 7 Real-Time PCR System. MicroRNA expression levels were normalized to two different internal control small RNAs (RNU48 and U6 snRNA) obtaining similar results. The comparative threshold cycle method was used to calculate the relative microRNA expression. Statistical analysis of microRNAs differentially expressed among samples was carried out by means of StatMiner Software V5.0 (Integromics Inc., Waunakee, WI) through the use of paired *t*-test (*p* < 0.05).

### 2.7. Pathway Analysis

The candidate proteins, microRNAs, and genes were used as input in the GePS (Genomatix Pathway System, Release 2.7.1, Genomatix Genome Analyzer v3.30126) in order to investigate the connections between the 8 proteins, 22 microRNAs, and 7 genes. No extension of the network was performed since our approach was to identify connections between the above-mentioned entities without external influence.

### 2.8. Bioinformatics and Statistical Analysis

Data reported in this paper are the mean ± S.D. of at least three independent experiments, each performed in triplicate. Unless otherwise stated, statistical analysis was performed by the Student *t*-test and experimental data elaborated by means of the GraphPad Prism 5 software (GraphPad Software for Science, San Diego). Unsupervised clustering and heat maps were generated in Gene-E (version 3.0.238, http://www.broadinstitute.org/cancer/software/GENE-E/) using microRNA expression levels as input. In Gene-E the one minus Pearson correlation method was used for clustering and the average linkage as a linkage method.

## 3. Results

### 3.1. Proteins and Genes Characterizing hMB-SLCs

**Figure 1:**
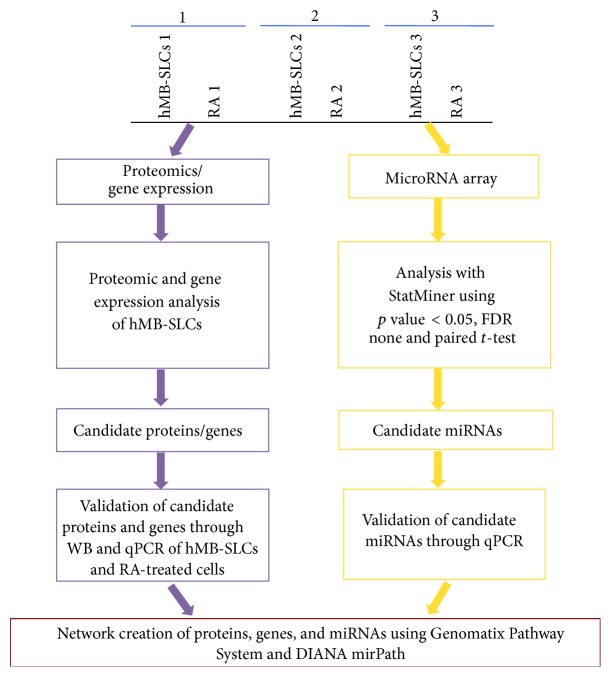
Experimental design and analysis of hMB-SLCs. Three biological cell replicates obtained by untreated and RA-treated cells were compared for proteome, genes, and microRNAs expression patterns.

**Figure 2:**
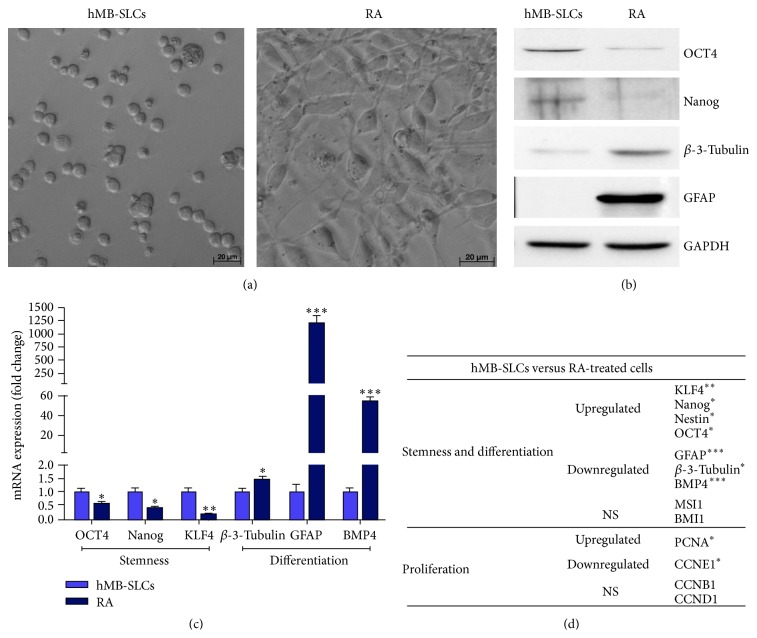
Morphology and characteristics of hMB-SLCs. (a) hMB-SLCs were cultured for 48 h in the absence or in the presence of 8 *μ*M RA which induces SLCs differentiation, highlighted by morphological changes and cell adhesion. (b) Western blot analysis confirmed that 48 h exposure of hMB-SLCs to 8 *μ*M RA induced a strong reduction in the stemness markers Nanog and OCT4 while increasing the differentiation markers, *β*-3-Tubulin and GFAP. (c) qPCR analysis of* OCT4*,* Nanog*,* KLF4*, *β-3-Tubulin*,* GFAP,* and* BMP4* was conducted before and after RA treatment. mRNA expression levels are indicated as fold changes with respect to hMB-SLCs. (d) Gene expression of stemness, differentiation, and proliferation markers in hMB-SLCs with respect to RA-treated cells. *∗* denotes *p* < 0.05 versus hMB-SLCs, *∗∗* denotes *p* < 0.01 versus hMB-SLCs, and *∗∗∗* denotes *p* < 0.001 versus hMB-SLCs.

To investigate proteins and genes features of hMB-SLCs, three biological cell replicates obtained by untreated and RA-treated hMB-SLCs were analyzed (Figure 1). As shown in Figure 2(a), 48 h after RA treatment hMB-SLCs displayed typical morphological changes of differentiated cells, compared to control cells. In accordance with morphological changes, also the expression of stemness and differentiation specific markers was up- or downregulated in control and RA-treated cells, respectively, both at protein (Figure 2(b)) and transcript (Figures 2(c) and 2(d)) level.

In particular, among stemness genes* Nanog*,* Kruppel-Like Factor 4* (*KLF4*),* Nestin,* and* Octamer Binding Transcription Factor 4* (*OCT4*) characterized stem cells analyzed while* BMP4*, *β-3-Tubulin,* and* GFAP* characterized RA-treated cells (Figures 2(c) and 2(d)). Among proliferation genes, the two markers significantly modulated at the transcript level were* Proliferating Cell Nuclear Antigen* (*PCNA*) and* Cyclin E1* (*CCNE1*), respectively, up- and downregulated, while* Cyclin B1* and* Cyclin D1* (*CCNB1*,* CCND1*) expression was not modified (Figure 2(d)).

### 3.2. MicroRNAs Patterns in hMB-SLCs

The three biological cell replicates were further investigated to highlight microRNA patterns (Figure 1).

**Figure 3:**
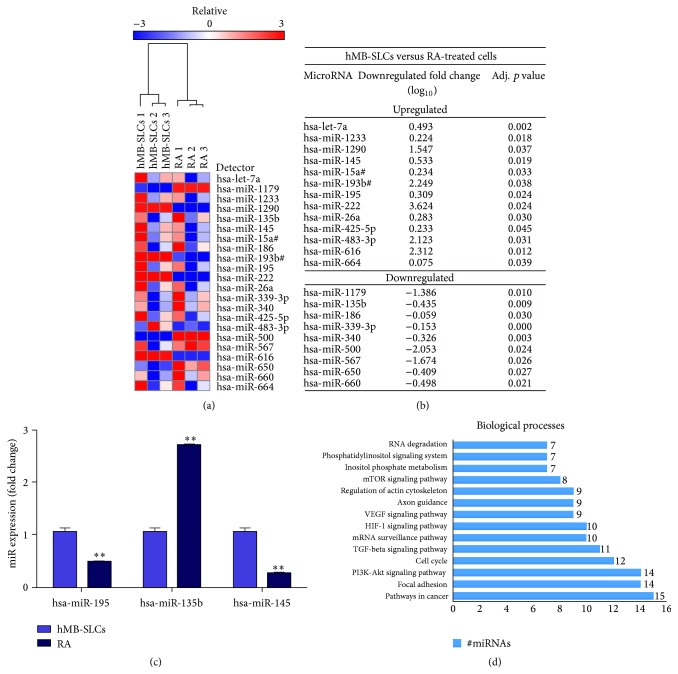
MicroRNA profiling of hMB-SLCs and RA-treated cells and qPCR validation. (a) Heatmap with unsupervised hierarchical clustering was obtained for hMB-SLCs and their differentiated counterparts. Dendrogram which represents the results of the hierarchical clustering analysis distinguishes hMB-SLCs from RA-treated ones. (b) Significantly up- or downregulated microRNAs in hMB-SLCs versus RA-treated cells with the respective fold change and *p* values are reported. (c) Single qPCR analysis of miR-195, miR-135b, and miR-145 was conducted. MicroRNAs expression levels are expressed as fold change with respect to hMB-SLCs. *∗∗* denotes *p* < 0.01 versus hMB-SLCs. (d) Biological processes ranked according to the number of microRNAs involved in each process are assigned as in KEGG pathway.

Our analysis revealed 22 microRNAs (Figures 3(a) and 3(b)) differentially expressed after 48 h of RA treatment. In order to endorse our results, we focused on 3 out of 22 microRNAs known to be involved in cell proliferation, stemness maintenance, and tumor invasiveness for qPCR validation (Figure 3(c)). Then, by using mirPath (http://diana.imis.athena-innovation.gr/DianaTools/index.php?r=mirpath/index) we were able to identify the molecular and biological functions mainly involved in the RA-induced differentiation process (Figure 3(d) and Supplementary Table  1 in Supplementary Material available online at http://dx.doi.org/10.1155/2016/2683042).

### 3.3. MicroRNA Targets

MicroRNAs are known to regulate gene expression at the posttranscriptional level and have multitarget characteristics, being able to control groups of genes; thus, we focused on the identification of all possible target genes of the 22 differentially expressed microRNAs (Figures 3(a) and 3(b)) by using miRTarBase (Supplementary Table  2). Moreover, since microRNAs negatively regulate mRNAs, we expected that increased microRNA expression would be associated with targets that decreased in expression and vice versa; among them, we deeply analyzed those that could have a role in the regulation of proliferation and/or differentiation of hMB-SLCs. In particular, miR-195 upregulated after RA treatment targeted* CCNE1* that was accordingly downregulated after RA-treatment. In addition also miR-145, which belongs to the same family of miR-195, was upregulated in RA-treated cells. Its targets, such as* KLF4* and* OCT4* [15], were downregulated after RA-treatment. Conversely miR-135b, which was downregulated after RA-treatment, targeted* Midline 1 Ring Finger Protein* (*MID1*), upregulated in RA-treated hMB-SLCs (Table 1).

### 3.4. MicroRNAs, Genes, and Proteomic Networks

**Figure 4:**
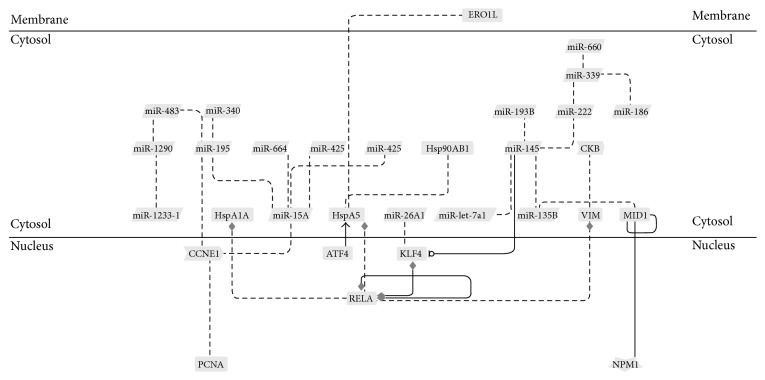
Interaction network of microRNAs, proteins, and genes in hMB-SLCs by means of GePS analysis.

**Figure 5:**
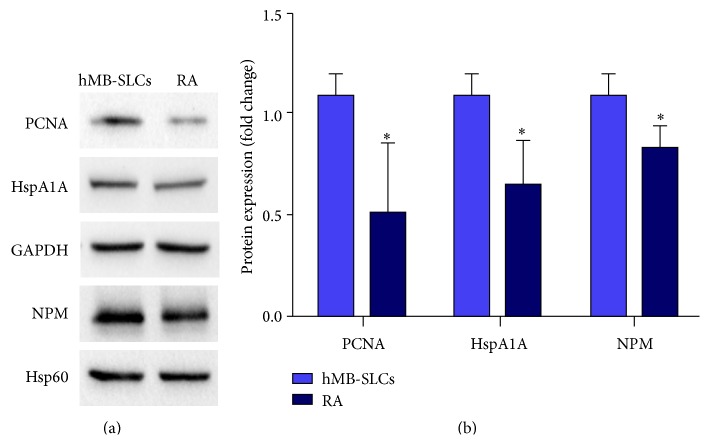
WB validation. (a) Western blot analysis of NPM, PCNA, and HspA1A in hMB-SLCs and in 48 h RA-treated cells. Hsp60 and GAPDH were used as control for equal protein load. (b) Densitometric analysis confirmed the significant differential expression observed by proteomic analysis. The protein expression, normalized to Hsp60 expression for NPM and GAPDH expression for the other proteins, represents the mean ± SEM from three independent experiments. *∗* denotes *p* < 0.05 versus hMB-SLCs.

**Figure 6:**
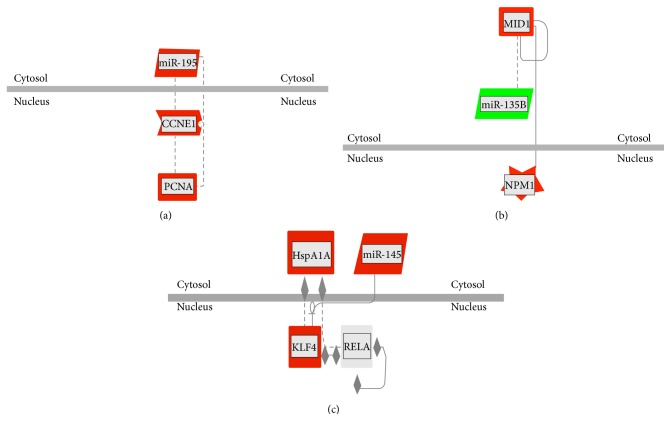
Subnetworks of microRNAs, proteins, and genes putatively involved in the maintenance of hMB-SLCs proliferation. (a) miR-195 has direct connections to* CCNE1* that targets PCNA. (b) NPM1 (NPM) interacts with* MID1* that targets the oncogenic microRNA, miR-135b. (c) miR-145 regulates the stemness marker* KLF4* that interacts both with p65 and with the heat-shock protein HspA1A.

Recently, we investigated the proteomic profile of hMB-SLCs and their RA-differentiated counterparts applying a label-free quantitative proteomic analysis able to maximize the identification capacities of the statistically differential spectral features [8]. Thus, to identify networks of microRNAs, genes, and proteins that could be involved in hMB-SLCs maintenance, we considered the previously described proteomic profile of hMB-SLCs [8] together with the new data reported in this paper as input in a GePS analysis (Figure 4). In order to endorse our proteomic results, by means of western blotting we validated the 3 out of 68 proteins (Figure 5) derived from [8] that connected with significantly deregulated microRNAs and genes, feature of the analyzed stem cells. In particular, we concentrated on three subnetworks that could be interestingly involved in the maintenance of hMB-SLCs proliferation (Figures 6(a), 6(b), and 6(c)). In Figure 6(a) a network including miR-195 and its proliferative target* CCNE1* is shown. In addition to* CCNE1* also PCNA, another known target of miR-195 [16] is included in the network. The second network (Figure 6(b)) comprises miR-135b, its direct target* MID1,* and Nucleophosmin (NPM), an important player in cell proliferation and apoptosis [17]. Finally the third network shows the link among the tumor suppressive microRNA, miR-145 [18],* KLF4*, p65, and the Heat Shock 70 kDa Protein (HspA1A) (Figure 6(c)).

## 4. Discussion

MB is under continuous study in order to define the signaling pathways involved in its growth and progression and in order to avoid overtreatment and therapy related side effects on the developing brain [19]. However the existence of cancer stem cells, which represent a resistant subpopulation, makes both tumor treatment and eradication more difficult, raising a high interest in the research of signaling that drives those cells.

In this study, we focused on the analysis of miRNome and genes expression profile of hMB-SLCs with respect to their RA-differentiated counterparts in order to identify components that are specifically up- or downregulated and thus may play a role in their maintenance. MicroRNAs indeed are involved in the regulation of mRNA and protein expression levels, concordantly modulated as shown in the present study, even though they can also intervene in other important regulatory mechanisms. Therefore we used the GePS analysis in order to identify specific pathways that support proliferation and survival of hMB-SLCs or conversely sustain hMB-SLCs differentiation in a more mature and less aggressive phenotype.

In a recent study we revealed 68 proteins specific for hMB-SLCs [8] and in this study we extended the analysis to microRNAs and genes and identified 22 microRNAs (Figures 3(a) and 3(b)) that were significantly modulated after RA-treatment. First of all, in order to endorse our results, we selected some biologically relevant microRNAs and proteins and conducted specific single qPCR (Figure 3(c)) and western blotting analysis (Figures 5(a) and 5(b)) to confirm the quality of the data obtained by using high-throughput technology screening, as MS spectrometry [8] and microRNAs arrays. Since our results indicated a good correlation among the different technologies, we used mirPath (Figure 3(d) and Supplementary Table  1) and GePS software (Figure 4) in order to define the biological processes in which these differentially expressed microRNAs and validated proteins were both involved. The most interesting among the significant biological processes that resulted from our analysis referred to cancer cell activated pathways, such as mTOR, TGF*β*, and PI3K-Akt signaling, cell cycle regulation, cytoskeleton remodeling, and energy metabolism. All these biological events are already known to be deregulated not only in cancer cells but also in cancer stem cells.

Subsequently, since our aim was the identification of networks involved in hMB-SLCs maintenance, we used differentially expressed microRNAs and proteins as input and analyzed their relationship by using the GePS analysis software (Figure 4). Focusing our attention on the network we obtained, we uncovered three subnetworks that could be interestingly involved in the maintenance of hMB-SLCs proliferation (Figures 6(a), 6(b), and 6(c)). As shown in Figure 6(a), miR-195,* CCNE1*, and PCNA are involved in the first subnetwork. CCNE1 is a key regulator of cell cycle promoting the progression to the G1 phase and the entry in the S phase [20, 21]. Together with CCND1, which leads to the hyperphosphorylation of the tumor suppressor protein retinoblastoma (pRb) and to its dissociation of the E2 promoter-binding protein dimerization partners (E2F) from the pRb/E2F complex, CCNE1 is considered to be a key oncogene and is overexpressed in breast, liver, lung, and brain cancers [22, 23]. However, despite its role as an oncogene, only recently it has been proposed in human glioma cells a mechanism for the regulation of CCNE1 activity that involves the miR-195 and leads to the reduction of pRb phosphorylation and to the downregulation of the proliferative marker PCNA [16]. In our model, miR-195 was significantly upregulated after hMB-SLCs differentiation, in contrast to its direct target CCNE1, whose expression was significantly reduced. As a consequence of the inhibition of hMB-SLCs proliferation, also PCNA expression was strongly decreased. Thus, our results point out that the mechanism of CCNE1 modulation by miR-195 and the subsequent reduction of PCNA may be also involved in the regulation of hMB-SLCs proliferation, suggesting that the inhibition and/or activation of one or more players of this network could be of strong interest in the clinical management of hMB-SLCs.

The second subnetwork (Figure 6(b)) included miR-135b,* MID1,* and NPM (NPM1). In a recent high throughput study conducted on 6 different pediatric SLCs, not only was miR-135b significantly upregulated in the SLCs fraction compared to each non-SLCs reference fraction but also its silencing strongly inhibited their ability of self-renewal [24]. In addition, this microRNA has been shown to be overexpressed in hMB compared to normal cerebellum [25] and to correlate with poor prognosis and degree of malignancy in a number of different tumors, such as colon cancer, ependymoma, and hepatocellular carcinoma [26–29]. One of its most interesting targets with the aim of unraveling tumor biology is the microtubule-associated ubiquitin E3 ligase MID1, whose downregulation in human solid tumors has been associated with a more aggressive phenotype and with an increased invasiveness [30]. Furthermore a direct physical interaction between MID1 and NPM has been lately reported [31], a multifunctional nucleolar phosphoprotein required for the assembly of ribosomes, that has also been described as a MB resistance marker and whose expression is correlated to the ability of MB to survive in unfavorable growth conditions [19]. Interestingly our results showed a significant reduction of both miR-135b and NPM expression after RA-induced differentiation associated with a strong upregulation of MID1, highlighting their negative correlation compared to this gene and suggesting that also in this case the pharmacological regulation of these players could be very useful in order to control MB growth.

Finally the third subnetwork (Figure 6(c)) consisted of four different players, miR-145,* KLF4*, p65, and HspA1A. MiR-145 has been recently described as a dominant player in the differentiation process of human embryonic stem cells (hESCs). In these cells, miR-145 significantly increased during differentiation and acted through the posttranscriptional downregulation of OCT4, SOX2, and KLF4 [15]. As described in hESCs, our results confirmed that also in hMB-SLCs there was a significant increase of miR-145 after 48 h of RA-treatment and a strong decrease of some of these stemness markers, as shown in Figures 2(b), 2(c), and 2(d). In addition KLF4, a well-known pluripotency factor [32] which is implicated in glioblastoma stem cells proliferation, migration, and invasion [33] has also been demonstrated to physically interact with p65, a member of the NF-*κ*B family of transcription factors involved in the regulation of a wide variety of biological responses and with a pivotal role in oncogenesis [34], in the induction of a macrophage-mediated proinflammatory pathway activation [35]. Very recently we have shown that the NF-*κ*B complex was strongly activated in hMB-SLCs and hypothesized its connection with the molecular chaperone HspA1A, which increased in hMB-SLCs and significantly decreased after RA-induced differentiation [8]. Thus, this third network corroborates our recent hypothesis and underlines the fact that this microRNA, repressing some core pluripotency factors such as OCT4 and KLF4, is crucially involved in the modulation of the differentiation pathway progression of cancer stem cells.

## 5. Conclusions

Taken together, our results report the analysis of microRNAs, genes, and proteins that are significantly regulated in hMB-SLCs with respect to their RA-differentiated counterparts. Many of these microRNAs through a direct mRNA interaction may act on different proteins, such as NPM, PCNA, p65, and HspA1A, with the creation of networks involved in the induction of aberrant cell growth and proliferation other than programmed cell death resistance linked to SLCs features. In conclusion, our findings could open new perspectives for prognostic and therapeutic intervention on MB.

## Supplementary Material

Supplementary Material DIANA mirPath
The 22 differentially expressed microRNAs were used as input in the DIANA mirPath (http://diana.imis.athena-innovation.gr/DianaTools/index.php?r=mirpath/index) for the investigation of their molecular and biological functions. A p value < 0.05 was considered as significant and the result is reported in Supplementary Table 1. In detail the number of microRNAs and genes involved in each function is listed.

## Figures and Tables

**Table 1:** Differentially regulated interaction between microRNAs and genes.

MicroRNA	MicroRNA fold change (Log_10_)	Target gene	mRNA RQ
hsa-miR-135b	−0.435	MID1	8.5
hsa-miR-145	0.533	KLF4	0.198
hsa-miR-195	0.309	CCNE1	0.445
