# Supplementary material for: MicroRNAs-Proteomic Networks Characterizing Human Medulloblastoma-SLCs
Source: Stem Cells Int. 2016 Jan 6;2016:2683042. doi: 10.1155/2016/2683042 (PMC4736593; doi:10.1155/2016/2683042)
Supplement: Supplementary file 1 — Supplementary Material DIANA mirPath The 22 differentially expressed microRNAs were used as input in the DIANA mirPath (http://diana.imis.athena-innovation.gr/DianaTools/index.php?r=mirpath/index) for the investigation of their molecular and biological functions. A p value < 0.05 was considered as significant and the result is reported in Supplementary Table 1. In detail the number of microRNAs and genes involved in each function is listed. [file 2683042.f1.pdf]

Supplementary Table 1. microRNAs related pathways using mirPath

**KEGG pathway**	**P-value**	**#genes**	**#microRNAs**
Prostate cancer	2,91E-16	33	11
Pathways in cancer	1,65E-08	70	15
Colorectal cancer	2,37E-08	22	14
Hepatitis B	5,12E-08	41	13
Melanoma	6,23E-08	24	11
Bladder cancer	1,80E-07	17	8
Glioma	8,89E-07	23	11
Inositol phosphate metabolism	1,33E-04	16	7
Focal adhesion	1,70E-04	37	14
Non-small cell lung cancer	3,88E-04	18	8
TGF-beta signaling pathway	4,60E-04	18	11
mRNA surveillance pathway	4,60E-04	20	10
mTOR signaling pathway	4,60E-04	15	8
Pancreatic secretion	1,18E-03	19	8
Salmonella infection	1,24E-03	18	9
Acute myeloid leukemia	1,67E-03	18	11
Cell cycle	1,68E-03	27	12
Cholinergic synapse	2,18E-03	24	10
Insulin signaling pathway	2,69E-03	25	9
HIF-1 signaling pathway	2,98E-03	21	10
Pancreatic cancer	3,23E-03	22	12
VEGF signaling pathway	3,62E-03	14	9
Phosphatidylinositol signaling system	5,03E-03	18	7
Hepatitis C	5,90E-03	23	10
Epithelial cell signaling in Helicobacter pylori infection	5,90E-03	14	7

RNA degradation	6,56E-03	15	7
Shigellosis	7,26E-03	14	8
Salivary secretion	7,59E-03	17	7
Chronic myeloid leukemia	8,05E-03	22	12
Endometrial cancer	8,10E-03	17	10
Melanogenesis	8,61E-03	19	8
Gastric acid secretion	9,72E-03	15	8
Chemokine signaling pathway	1,05E-02	31	11
Alzheimer's disease	1,19E-02	28	11
PI3K-Akt signaling pathway	1,30E-02	64	14
Fc epsilon RI signaling pathway	1,33E-02	14	9
Axon guidance	1,35E-02	23	9
Regulation of actin cytoskeleton	1,66E-02	36	9
Renal cell carcinoma	1,77E-02	15	7
Dorso-ventral axis formation	1,77E-02	6	4
HTLV-I infection	1,98E-02	40	16
Serotonergic synapse	1,98E-02	19	8
Oocyte meiosis	2,30E-02	22	12
T cell receptor signaling pathway	2,35E-02	19	11
Circadian rhythm	2,35E-02	7	5
Adherens junction	2,48E-02	14	8
Endocrine and other factor-regulated calcium reabsorption	2,48E-02	11	8
Fanconi anemia pathway	2,66E-02	11	7
Dopaminergic synapse	2,74E-02	23	10
Viral myocarditis	2,92E-02	13	10
Pertussis	2,92E-02	14	7

Vasopressin-regulated water reabsorption	2,92E-02	10	5
Neurotrophin signaling pathway	3,30E-02	27	11
NOD-like receptor signaling pathway	3,30E-02	12	5
Progesterone-mediated oocyte maturation	4,13E-02	15	10
Chagas disease (American trypanosomiasis)	4,39E-02	18	11

Supplementary Table 2

miRTarBase ID	microRNA	Species (micro RNA)	Target Gene	Target Gene (Entrez ID)	Species (Target Gene)	Experiments	Support Type	References (PMID)
MIRT000417	hsa-let-7a-5p	Homo sapiens	MYC	4609	Homo sapiens	Western blot	Functional MTI	20033209
MIRT000417	hsa-let-7a-5p	Homo sapiens	MYC	4609	Homo sapiens	qRT-PCR//Western blot	Functional MTI	16651716
MIRT000463	hsa-let-7a-5p	Homo sapiens	NKIR AS2	28511	Homo sapiens	ELISA//qRT-PCR//Luciferase reporter assay//Western blot	Functional MTI	20351193
MIRT000708	hsa-let-7a-5p	Homo sapiens	ITGB3	3690	Homo sapiens	qRT-PCR//Luciferase reporter assay//Western blot	Functional MTI	18679415
MIRT001853	hsa-let-7a-5p	Homo sapiens	NF2	4771	Homo sapiens	Luciferase reporter assay//Microarray//qRT-PCR//Western blot//Reporter assay;Other	Functional MTI	17220301
MIRT001854	hsa-let-7a-5p	Homo sapiens	NRAS	4893	Homo sapiens	Western blot	Functional MTI	19110058
MIRT001854	hsa-let-7a-5p	Homo sapiens	NRAS	4893	Homo sapiens	Luciferase reporter assay//Microarray	Functional MTI	15766527
MIRT001855	hsa-let-7a-5p	Homo sapiens	KRAS	3845	Homo sapiens	Luciferase reporter assay//Microarray	Functional MTI	15766527
MIRT001855	hsa-let-7a-5p	Homo sapiens	KRAS	3845	Homo sapiens	Western blot	Functional MTI	20033209
MIRT001855	hsa-let-7a-5p	Homo sapiens	KRAS	3845	Homo sapiens	qRT-PCR//Western blot	Functional MTI	16651716
MIRT001936	hsa-let-7a-5p	Homo sapiens	PRDM 1	639	Homo sapiens	Luciferase reporter assay	Functional MTI	18583325
MIRT001936	hsa-let-7a-5p	Homo sapiens	PRDM 1	639	Homo sapiens	Immunohistochemistry//Luciferase reporter assay//qRT-PCR//Western blot	Functional MTI	20651244
MIRT002077	hsa-let-7a-5p	Homo sapiens	TRIM7 1	131405	Homo sapiens	Luciferase reporter assay//Reporter assay	Functional MTI	17890240
MIRT002077	hsa-let-7a-5p	Homo sapiens	TRIM7 1	131405	Homo sapiens	Luciferase reporter assay	Functional MTI	18829576
MIRT002305	hsa-let-7a-5p	Homo sapiens	RAVE R2	55225	Homo sapiens	Immunoprecipitaion//Luciferase reporter assay//Western blot	Functional MTI	17637574
MIRT002323	hsa-let-7a-5p	Homo sapiens	HMGA 2	8091	Homo sapiens	qRT-PCR//Western blot//Luciferase reporter assay	Functional MTI	17600087
MIRT002323	hsa-let-7a-5p	Homo sapiens	HMGA 2	8091	Homo sapiens	Luciferase reporter assay	Functional MTI	18083101
MIRT002323	hsa-let-7a-5p	Homo sapiens	HMGA 2	8091	Homo sapiens	Luciferase reporter assay	Functional MTI	17322030
MIRT002323	hsa-let-7a-5p	Homo sapiens	HMGA 2	8091	Homo sapiens	Luciferase reporter assay	Functional MTI	18413822
MIRT002323	hsa-let-7a-5p	Homo sapiens	HMGA 2	8091	Homo sapiens	Luciferase reporter assay//Western blot//qRT-PCR	Functional MTI	19179606
MIRT003152	hsa-let-7a-5p	Homo sapiens	HMGA 1	3159	Homo sapiens	Luciferase reporter assay//qRT-PCR	Functional MTI	19179606
MIRT003522	hsa-let-7a-5p	Homo sapiens	AGO4	192670	Homo sapiens	Luciferase reporter assay	Functional MTI	20144220

MIRT004321	hsa-let-7a-5p	Homo sapiens	UHRF 2	115426	Homo sapiens	qRT-PCR//Western blot//Luciferase reporter assay	Functional MTI	19818775
MIRT004346	hsa-let-7a-5p	Homo sapiens	DICER 1	23405	Homo sapiens	Luciferase reporter assay//Western blot//Northern blot	Functional MTI	18700235
MIRT004471	hsa-let-7a-5p	Homo sapiens	HRAS	3265	Homo sapiens	Luciferase reporter assay	Functional MTI	18083101
MIRT004520	hsa-let-7a-5p	Homo sapiens	IGF2	3481	Homo sapiens	Luciferase reporter assay	Functional MTI	17974952
MIRT004801	hsa-let-7a-5p	Homo sapiens	LIN28 A	79727	Homo sapiens	B-globin reporter assay//Luciferase reporter assay//Microarray	Functional MTI	16495412
MIRT005292	hsa-let-7a-5p	Homo sapiens	CASP3	836	Homo sapiens	Luciferase reporter assay//Western blot	Functional MTI	18758960
MIRT005420	hsa-let-7a-5p	Homo sapiens	IL6	3569	Homo sapiens	Luciferase reporter assay//qRT-PCR//Western blot	Functional MTI	19878981
MIRT005477	hsa-let-7a-5p	Homo sapiens	E2F2	1870	Homo sapiens	Luciferase reporter assay//qRT-PCR//Western blot	Functional MTI	20418948
MIRT005478	hsa-let-7a-5p	Homo sapiens	CCND 2	894	Homo sapiens	Luciferase reporter assay//qRT-PCR//Western blot	Functional MTI	20418948
MIRT032506	hsa-let-7a-5p	Homo sapiens	CDKN 1A	1026	Homo sapiens	Western blot	Functional MTI	19818775
MIRT035534	hsa-let-7a-5p	Homo sapiens	HAS2	3037	Homo sapiens	Luciferase reporter assay	Functional MTI	22871741
MIRT035541	hsa-let-7a-5p	Homo sapiens	EGFR	1956	Homo sapiens	Luciferase reporter assay	Functional MTI	23032975
MIRT003596	hsa-miR-135b-5p	Homo sapiens	BGLA P	632	Homo sapiens	qRT-PCR	Non-Functional MTI (Weak)	19795981
MIRT003597	hsa-miR-135b-5p	Homo sapiens	RUNX 2	860	Homo sapiens	qRT-PCR	Non-Functional MTI (Weak)	19795981
MIRT005440	hsa-miR-135b-5p	Homo sapiens	KLF4	9314	Homo sapiens	Luciferase reporter assay//Microarray//qRT-PCR	Functional MTI	20981674
MIRT005442	hsa-miR-135b-5p	Homo sapiens	MAFB	9935	Homo sapiens	Luciferase reporter assay//Microarray//qRT-PCR	Functional MTI	20981674
MIRT007298	hsa-miR-135b-5p	Homo sapiens	CASR	846	Homo sapiens	Western blot	Functional MTI	23340180
MIRT000305	hsa-miR-145-5p	Homo sapiens	BNIP3	664	Homo sapiens	Luciferase reporter assay//qRT-PCR//Western blot	Functional MTI	20332243
MIRT000307	hsa-miR-145-5p	Homo sapiens	SOX2	6657	Homo sapiens	FACS//Flow//GFP reporter assay//In situ hybridization//Luciferase reporter assay//qRT-PCR//Reporter assay;Western blot;qRT-PCR;Other	Functional MTI	19409607
MIRT000307	hsa-miR-145-5p	Homo sapiens	SOX2	6657	Homo sapiens	Luciferase reporter assay	Functional MTI	23541921
MIRT000308	hsa-miR-145-5p	Homo sapiens	KLF4	9314	Homo sapiens	FACS//Flow//GFP reporter assay//In situ hybridization//Luciferase reporter assay//qRT-PCR//Reporter assay;Western blot;qRT-PCR	Functional MTI	19409607
MIRT000426	hsa-miR-145-5p	Homo sapiens	MUC1	4582	Homo sapiens	Luciferase reporter assay//Western blot	Functional MTI	20407606
MIRT000426	hsa-miR-145-5p	Homo sapiens	MUC1	4582	Homo sapiens	Immunohistochemistry//Luciferase reporter assay//qRT-PCR//Western blot//Reporter assay	Functional MTI	19996288

MIRT000457	hsa-miR-145-5p	Homo sapiens	MYO6	4646	Homo sapiens	qRT-PCR//Luciferase reporter assay//Western blot	Functional MTI	20353999
MIRT006494	hsa-miR-145-5p	Homo sapiens	ITGB8	3696	Homo sapiens	Immunofluorescence//Luciferase reporter assay//qRT-PCR//GFP reporter assay//In situ hybridization//Microarray//Western blot	Functional MTI	21701675
MIRT000626	hsa-miR-145-5p	Homo sapiens	STAT1	6772	Homo sapiens	qRT-PCR//Luciferase reporter assay//Western blot//Microarray	Functional MTI	20098684
MIRT000627	hsa-miR-145-5p	Homo sapiens	YES1	7525	Homo sapiens	qRT-PCR//Luciferase reporter assay//Western blot//Microarray	Functional MTI	20098684
MIRT000627	hsa-miR-145-5p	Homo sapiens	YES1	7525	Homo sapiens	Reporter assay;Microarray	Functional MTI	21351259
MIRT000676	hsa-miR-145-5p	Homo sapiens	CBFB	865	Homo sapiens	qRT-PCR//Luciferase reporter assay//Microarray//Reporter assay;Other	Functional MTI	19915607
MIRT000677	hsa-miR-145-5p	Homo sapiens	PPP3CA	5530	Homo sapiens	qRT-PCR//Luciferase reporter assay//Microarray//Reporter assay;Other	Functional MTI	19915607
MIRT000678	hsa-miR-145-5p	Homo sapiens	CLINT1	9685	Homo sapiens	qRT-PCR//Luciferase reporter assay//Microarray//Reporter assay;Other	Functional MTI	19915607
MIRT000731	hsa-miR-145-5p	Homo sapiens	IRS1	3667	Homo sapiens	Luciferase reporter assay//Northern blot//qRT-PCR//Western blot//Reporter assay;Other	Functional MTI	17827156
MIRT000731	hsa-miR-145-5p	Homo sapiens	IRS1	3667	Homo sapiens	qRT-PCR//Western blot	Functional MTI	19391107
MIRT000731	hsa-miR-145-5p	Homo sapiens	IRS1	3667	Homo sapiens	Luciferase reporter assay	Functional MTI	22431718
MIRT000731	hsa-miR-145-5p	Homo sapiens	IRS1	3667	Homo sapiens	Luciferase reporter assay//Western blot	Functional MTI	23201159
MIRT000732	hsa-miR-145-5p	Homo sapiens			Homo sapiens	Luciferase reporter assay	Functional MTI	15131085
MIRT006332	hsa-miR-145-5p	Homo sapiens	IRS2	8660	Homo sapiens	Luciferase reporter assay	Functional MTI	22431718
MIRT006215	hsa-miR-145-5p	Homo sapiens	VEGFA	7422	Homo sapiens	Luciferase reporter assay//qRT-PCR//Western blot	Functional MTI	22472569
MIRT003543	hsa-miR-145-5p	Homo sapiens	FSCN1	6624	Homo sapiens	Luciferase reporter assay//qRT-PCR//Western blot	Functional MTI	20160723
MIRT003543	hsa-miR-145-5p	Homo sapiens	FSCN1	6624	Homo sapiens	Luciferase reporter assay//Microarray//qRT-PCR//Western blot//Reporter assay;Western blot;qRT-PCR;Microarray	Functional MTI	21351259
MIRT003543	hsa-miR-145-5p	Homo sapiens	FSCN1	6624	Homo sapiens	Luciferase reporter assay//qRT-PCR//Western blot//Reporter assay	Functional MTI	20198616
MIRT003543	hsa-miR-145-5p	Homo sapiens	FSCN1	6624	Homo sapiens	Reporter assay	Functional MTI	21258769
MIRT004290	hsa-miR-145-5p	Homo sapiens	MYC	4609	Homo sapiens	qRT-PCR//Luciferase reporter assay//Western blot	Functional MTI	19202062
MIRT004290	hsa-miR-145-5p	Homo sapiens	MYC	4609	Homo sapiens	Luciferase reporter assay//qRT-PCR//Western blot	Functional MTI	21092188
MIRT004496	hsa-miR-145-5p	Homo sapiens	FLI1	2313	Homo sapiens	qRT-PCR//Luciferase reporter assay//Western blot	Functional MTI	20382729
MIRT004496	hsa-miR-145-5p	Homo sapiens	FLI1	2313	Homo sapiens	Luciferase reporter assay//Northern blot//qRT-PCR//Western blot	Functional MTI	20737575
MIRT004496	hsa-miR-	Homo	FLI1	2313	Homo	Luciferase reporter assay//Western blot	Functional MTI	21217773

	145-5p	sapiens			sapiens			
MIRT004580	hsa-miR-145-5p	Homo sapiens			Homo sapiens	Luciferase reporter assay//qRT-PCR//Western blot	Functional MTI	20687965
MIRT004616	hsa-miR-145-5p	Homo sapiens	IFNB1	3456	Homo sapiens	ELISA//Luciferase reporter assay//qRT-PCR	Functional MTI	20130213
MIRT004748	hsa-miR-145-5p	Homo sapiens	TIRAP	114609	Homo sapiens	Immunoprecipitaion//Western blot//Communoprecipitaion	Functional MTI	19898489
MIRT004904	hsa-miR-145-5p	Homo sapiens	POU5F1	5460	Homo sapiens	FACS//Flow//GFP reporter assay//In situ hybridization//Luciferase reporter assay//qRT-PCR//Reporter assay;Western blot;qRT-PCR	Functional MTI	19409607
MIRT004904	hsa-miR-145-5p	Homo sapiens	POU5F1	5460	Homo sapiens	Luciferase reporter assay	Functional MTI	21496429
MIRT004904	hsa-miR-145-5p	Homo sapiens	POU5F1	5460	Homo sapiens	Luciferase reporter assay	Functional MTI	23541921
MIRT004931	hsa-miR-145-5p	Homo sapiens	IGF1R	3480	Homo sapiens	qRT-PCR//Western blot	Functional MTI	19391107
MIRT004938	hsa-miR-145-5p	Homo sapiens	KRT7	3855	Homo sapiens	Immunoblot//qRT-PCR	Non-Functional MTI (Weak)	19378336
MIRT005809	hsa-miR-145-5p	Homo sapiens	ROBO2	6092	Homo sapiens	In situ hybridization//Luciferase reporter assay//qRT-PCR//western blot//Reporter assay	Functional MTI	21276775
MIRT005810	hsa-miR-145-5p	Homo sapiens	SRGAP1	57522	Homo sapiens	In situ hybridization//Luciferase reporter assay//qRT-PCR//western blot	Functional MTI	21276775
MIRT005878	hsa-miR-145-5p	Homo sapiens	EIF4E	1977	Homo sapiens	Luciferase reporter assay//qRT-PCR//Western blot	Functional MTI	21092188
MIRT005879	hsa-miR-145-5p	Homo sapiens	CDK4	1019	Homo sapiens	Luciferase reporter assay//qRT-PCR//Western blot	Functional MTI	21092188
MIRT006317	hsa-miR-145-5p	Homo sapiens	SERPINE1	5054	Homo sapiens	Immunoblot//Luciferase reporter assay//qRT-PCR//Western blot	Functional MTI	22108519
MIRT006747	hsa-miR-145-5p	Homo sapiens	SWAP70	23075	Homo sapiens	Reporter assay;Microarray	Functional MTI	21351259
MIRT006747	hsa-miR-145-5p	Homo sapiens	SWAP70	23075	Homo sapiens	Reporter assay;Western blot;qRT-PCR	Functional MTI	21360565
MIRT006889	hsa-miR-145-5p	Homo sapiens	NEDD9	4739	Homo sapiens	Immunofluorescence//Immunohistochemistry//qRT-PCR//Western blot	Functional MTI	22869051
MIRT006889	hsa-miR-145-5p	Homo sapiens	NEDD9	4739	Homo sapiens	Luciferase reporter assay	Functional MTI	23355420
MIRT006899	hsa-miR-145-5p	Homo sapiens	PAK4	10298	Homo sapiens	Luciferase reporter assay//qRT-PCR//Western blot	Functional MTI	22766504
MIRT006903	hsa-miR-145-5p	Homo sapiens	DDX17	10521	Homo sapiens	Luciferase reporter assay//Northern blot//qRT-PCR//Western blot	Functional MTI	22876303
MIRT007094	hsa-miR-145-5p	Homo sapiens	ERG	2078	Homo sapiens	Luciferase reporter assay	Functional MTI	23480797
MIRT007107	hsa-miR-145-5p	Homo sapiens	NRAS	4893	Homo sapiens	Luciferase reporter assay//Western blot	Functional MTI	23201159
MIRT007180	hsa-miR-145-5p	Homo sapiens	ILK	3611	Homo sapiens	Luciferase reporter assay//qRT-PCR//Western blot	Functional MTI	23104321
MIRT007247	hsa-miR-145-5p	Homo sapiens	CTGF	1490	Homo sapiens	Luciferase reporter assay	Functional MTI	23390502

MIRT007248	hsa-miR-145-5p	Homo sapiens	SOCS7	30837	Homo sapiens	Luciferase reporter assay	Functional MTI	23392170
MIRT007257	hsa-miR-145-5p	Homo sapiens	MDM2	4193	Homo sapiens	Luciferase reporter assay	Functional MTI	22330136
MIRT007275	hsa-miR-145-5p	Homo sapiens	ADAM17	6868	Homo sapiens	Luciferase reporter assay	Functional MTI	23441135
MIRT007288	hsa-miR-145-5p	Homo sapiens	CDH2	1000	Homo sapiens	Luciferase reporter assay//Western blot	Functional MTI	22370644
MIRT007307	hsa-miR-145-5p	Homo sapiens	HDAC2	3066	Homo sapiens	Luciferase reporter assay	Functional MTI	23499894
MIRT021499	hsa-miR-145-5p	Homo sapiens	RTKN	6242	Homo sapiens	Reporter assay;Western blot;qRT-PCR;Other	Functional MTI	19360360
MIRT021501	hsa-miR-145-5p	Homo sapiens	F11R	50848	Homo sapiens	Reporter assay	Functional MTI	20818426
MIRT021501	hsa-miR-145-5p	Homo sapiens	F11R	50848	Homo sapiens	Reporter assay;Microarray	Functional MTI	21351259
MIRT021502	hsa-miR-145-5p	Homo sapiens	ARL6IP5	10550	Homo sapiens	Reporter assay;Microarray	Functional MTI	21351259
MIRT021503	hsa-miR-145-5p	Homo sapiens	AKR1B10	57016	Homo sapiens	Reporter assay;Microarray	Functional MTI	21351259
MIRT021504	hsa-miR-145-5p	Homo sapiens	C11orf65	160140	Homo sapiens	Reporter assay;Microarray	Functional MTI	21351259
MIRT021505	hsa-miR-145-5p	Homo sapiens	HLTF	6596	Homo sapiens	Reporter assay;Microarray	Functional MTI	21351259
MIRT021506	hsa-miR-145-5p	Homo sapiens	GMFB	2764	Homo sapiens	Reporter assay;Microarray	Functional MTI	21351259
MIRT021507	hsa-miR-145-5p	Homo sapiens	SERINC5	256987	Homo sapiens	Reporter assay;Microarray	Functional MTI	21351259
MIRT021508	hsa-miR-145-5p	Homo sapiens	MEST	4232	Homo sapiens	Reporter assay;Microarray	Functional MTI	21351259
MIRT021509	hsa-miR-145-5p	Homo sapiens	ALPPL2	251	Homo sapiens	Reporter assay;Microarray	Functional MTI	21351259
MIRT021510	hsa-miR-145-5p	Homo sapiens	NDRG2	57447	Homo sapiens	Reporter assay;Microarray	Functional MTI	21351259
MIRT021511	hsa-miR-145-5p	Homo sapiens	DTD1	92675	Homo sapiens	Reporter assay;Microarray	Functional MTI	21351259
MIRT021512	hsa-miR-145-5p	Homo sapiens	TPM3	7170	Homo sapiens	Reporter assay;Microarray	Functional MTI	21351259
MIRT021513	hsa-miR-145-5p	Homo sapiens	MAP2K6	5608	Homo sapiens	Reporter assay;Microarray	Functional MTI	21351259
MIRT021514	hsa-miR-145-5p	Homo sapiens	CEP19	84984	Homo sapiens	Reporter assay;Microarray	Functional MTI	21351259
MIRT021515	hsa-miR-145-5p	Homo sapiens	TPRG1	285386	Homo sapiens	Reporter assay;Microarray	Functional MTI	21351259
MIRT021516	hsa-miR-145-5p	Homo sapiens	GOLM1	51280	Homo sapiens	Reporter assay;Microarray	Functional MTI	21351259
MIRT021517	hsa-miR-145-5p	Homo sapiens	CCDC43	124808	Homo sapiens	Reporter assay;Microarray	Functional MTI	21351259

MIRT021518	hsa-miR-145-5p	Homo sapiens	MMP1	4312	Homo sapiens	Reporter assay;Microarray	Functional MTI	21351259
MIRT021519	hsa-miR-145-5p	Homo sapiens	PTP4A2	8073	Homo sapiens	Reporter assay;Microarray	Functional MTI	21351259
MIRT021520	hsa-miR-145-5p	Homo sapiens	TMEM9B	56674	Homo sapiens	Reporter assay;Microarray	Functional MTI	21351259
MIRT021521	hsa-miR-145-5p	Homo sapiens	MMP12	4321	Homo sapiens	Reporter assay;Microarray	Functional MTI	21351259
MIRT021522	hsa-miR-145-5p	Homo sapiens	MTMR14	64419	Homo sapiens	Reporter assay;Microarray	Functional MTI	21351259
MIRT021523	hsa-miR-145-5p	Homo sapiens	ALDH3A1	218	Homo sapiens	Reporter assay;Microarray	Functional MTI	21351259
MIRT021524	hsa-miR-145-5p	Homo sapiens	NDUFA4	4697	Homo sapiens	Reporter assay;Microarray	Functional MTI	21351259
MIRT021525	hsa-miR-145-5p	Homo sapiens	FAM3C	10447	Homo sapiens	Reporter assay;Microarray	Functional MTI	21351259
MIRT021526	hsa-miR-145-5p	Homo sapiens	LYPLA2	11313	Homo sapiens	Reporter assay;Microarray	Functional MTI	21351259
MIRT021527	hsa-miR-145-5p	Homo sapiens	FAM45A	404636	Homo sapiens	Reporter assay;Microarray	Functional MTI	21351259
MIRT021528	hsa-miR-145-5p	Homo sapiens	PIGF	5281	Homo sapiens	Reporter assay;Microarray	Functional MTI	21351259
MIRT021529	hsa-miR-145-5p	Homo sapiens	AP1G1	164	Homo sapiens	Reporter assay;Microarray	Functional MTI	21351259
MIRT021530	hsa-miR-145-5p	Homo sapiens	PHF17	79960	Homo sapiens	Reporter assay;Microarray	Functional MTI	21351259
MIRT021531	hsa-miR-145-5p	Homo sapiens	NIPSNAP1	8508	Homo sapiens	Reporter assay;Microarray	Functional MTI	21351259
MIRT021532	hsa-miR-145-5p	Homo sapiens	KREMEN1	83999	Homo sapiens	Reporter assay;Microarray	Functional MTI	21351259
MIRT021533	hsa-miR-145-5p	Homo sapiens	MMP14	4323	Homo sapiens	Reporter assay;Microarray	Functional MTI	21351259
MIRT021534	hsa-miR-145-5p	Homo sapiens	ABRACL	58527	Homo sapiens	Reporter assay;Microarray	Functional MTI	21351259
MIRT021535	hsa-miR-145-5p	Homo sapiens	MIXL1	83881	Homo sapiens	Reporter assay;Microarray	Functional MTI	21351259
MIRT021536	hsa-miR-145-5p	Homo sapiens	TSPAN6	7105	Homo sapiens	Reporter assay;Microarray	Functional MTI	21351259
MIRT021537	hsa-miR-145-5p	Homo sapiens	PODXL	5420	Homo sapiens	Reporter assay;Microarray	Functional MTI	21351259
MIRT021538	hsa-miR-145-5p	Homo sapiens	APH1A	51107	Homo sapiens	Reporter assay;Microarray	Functional MTI	21351259
MIRT021539	hsa-miR-145-5p	Homo sapiens	ABHD17C	58489	Homo sapiens	Reporter assay;Microarray	Functional MTI	21351259
MIRT035522	hsa-miR-145-5p	Homo sapiens	NANOG	79923	Homo sapiens	Luciferase reporter assay	Functional MTI	23541921
MIRT035535	hsa-miR-145-5p	Homo sapiens	MYO5A	4644	Homo sapiens	Luciferase reporter assay	Functional MTI	22895360

MIRT000280	hsa-miR-15a-5p	Homo sapiens	BMI1	648	Homo sapiens	Luciferase reporter assay//Western blot	Functional MTI	19903841
MIRT000282	hsa-miR-15a-5p	Homo sapiens	WNT3A	89780	Homo sapiens	Luciferase reporter assay	Functional MTI	18931683
MIRT000283	hsa-miR-15a-5p	Homo sapiens	MYB	4602	Homo sapiens	Luciferase reporter assay//Western blot	Functional MTI	18818396
MIRT000283	hsa-miR-15a-5p	Homo sapiens	MYB	4602	Homo sapiens	Luciferase reporter assay//Microarray//qRT-PCR	Functional MTI	21205891
MIRT000284	hsa-miR-15a-5p	Homo sapiens	CDC25A	993	Homo sapiens	Luciferase reporter assay	Functional MTI	18949056
MIRT000285	hsa-miR-15a-5p	Homo sapiens	CCND2	894	Homo sapiens	Luciferase reporter assay	Functional MTI	19549910
MIRT000815	hsa-miR-15a-5p	Homo sapiens	BCL2	596	Homo sapiens	Luciferase reporter assay	Functional MTI	17707831
MIRT000815	hsa-miR-15a-5p	Homo sapiens	BCL2	596	Homo sapiens	Luciferase reporter assay	Functional MTI	19478946
MIRT000815	hsa-miR-15a-5p	Homo sapiens	BCL2	596	Homo sapiens	Western blot	Functional MTI	20876285
MIRT000815	hsa-miR-15a-5p	Homo sapiens	BCL2	596	Homo sapiens	Luciferase reporter assay//qRT-PCR//Western blot	Functional MTI	16166262
MIRT000815	hsa-miR-15a-5p	Homo sapiens	BCL2	596	Homo sapiens	Western blot	Functional MTI	19903841
MIRT001227	hsa-miR-15a-5p	Homo sapiens	CCND1	595	Homo sapiens	qRT-PCR//Luciferase reporter assay//Western blot	Functional MTI	19591824
MIRT001227	hsa-miR-15a-5p	Homo sapiens	CCND1	595	Homo sapiens	Luciferase reporter assay	Functional MTI	19549910
MIRT001227	hsa-miR-15a-5p	Homo sapiens	CCND1	595	Homo sapiens	Luciferase reporter assay	Functional MTI	18931683
MIRT001228	hsa-miR-15a-5p	Homo sapiens	CCNE1	898	Homo sapiens	qRT-PCR//Luciferase reporter assay//Western blot	Functional MTI	19591824
MIRT001228	hsa-miR-15a-5p	Homo sapiens	CCNE1	898	Homo sapiens	Luciferase reporter assay	Functional MTI	19549910
MIRT001228	hsa-miR-15a-5p	Homo sapiens	CCNE1	898	Homo sapiens	immunohistochemistry//Luciferase reporter assay//Microarray//qRT-PCR	Functional MTI	19117988
MIRT002946	hsa-miR-15a-5p	Homo sapiens	DMTF1	9988	Homo sapiens	Luciferase reporter assay//Reporter assay	Functional MTI	15131085
MIRT003333	hsa-miR-15a-5p	Homo sapiens	BRCA1	672	Homo sapiens	Luciferase reporter assay	Functional MTI	19144710
MIRT003334	hsa-miR-15a-5p	Homo sapiens	AKT3	10000	Homo sapiens	Luciferase reporter assay	Functional MTI	23233752
MIRT003888	hsa-miR-15a-5p	Homo sapiens	CADM1	23705	Homo sapiens	Reporter assay//qRT-PCR	Functional MTI	18362358
MIRT004046	hsa-miR-15a-5p	Homo sapiens	UCP2	7351	Homo sapiens	Luciferase reporter assay//qRT-PCR	Functional MTI	21146880
MIRT004275	hsa-miR-15a-5p	Homo sapiens	VEGFA	7422	Homo sapiens	ELISA//Luciferase reporter assay	Functional MTI	18320040
MIRT004275	hsa-miR-15a-5p	Homo sapiens	VEGFA	7422	Homo sapiens	Luciferase reporter assay//qRT-PCR//Western blot	Functional MTI	23104180

MIRT004275	hsa-miR-15a-5p	Homo sapiens	VEGFA	7422	Homo sapiens	Luciferase reporter assay	Functional MTI	23233752
MIRT004680	hsa-miR-15a-5p	Homo sapiens	TSPYL2	64061	Homo sapiens	Luciferase reporter assay	Functional MTI	19478946
MIRT005552	hsa-miR-15a-5p	Homo sapiens	CHUK	1147	Homo sapiens	Luciferase reporter assay//qRT-PCR//Western blot	Functional MTI	20711193
MIRT005763	hsa-miR-15a-5p	Homo sapiens	TP53	7157	Homo sapiens	Immunoblot//Luciferase reporter assay	Functional MTI	21205967
MIRT006913	hsa-miR-15a-5p	Homo sapiens	IFNG	3458	Homo sapiens	Luciferase reporter assay//qRT-PCR	Functional MTI	22379033
MIRT006998	hsa-miR-15a-5p	Homo sapiens	PURA	5813	Homo sapiens	Luciferase reporter assay	Functional MTI	22835829
MIRT007090	hsa-miR-15a-5p	Homo sapiens	RECK	8434	Homo sapiens	GFP reporter assay//qRT-PCR	Functional MTI	23176145
MIRT003297	hsa-miR-186-5p	Homo sapiens	FOXO1	2308	Homo sapiens	Immunohistochemistry//Northern blot//qRT-PCR//Western blot	Functional MTI	20028871
MIRT004356	hsa-miR-186-5p	Homo sapiens	P2RX7	5027	Homo sapiens	qRT-PCR//Luciferase reporter assay	Functional MTI	18682393
MIRT005871	hsa-miR-186-5p	Homo sapiens	AKAP12	9590	Homo sapiens	Immunohistochemistry//Luciferase reporter assay//Microarray//qRT-PCR//Western blot	Functional MTI	20979053
MIRT007210	hsa-miR-186-5p	Homo sapiens	CSNK2A1	1457	Homo sapiens	Immunoblot//Luciferase reporter assay//qRT-PCR//Western blot	Functional MTI	23137536
MIRT000480	hsa-miR-193b-3p	Homo sapiens	CCND1	595	Homo sapiens	qRT-PCR//Luciferase reporter assay//Western blot//Microarray//Reporter assay;Western blot;qRT-PCR;Microarray;Other	Functional MTI	20304954
MIRT000480	hsa-miR-193b-3p	Homo sapiens	CCND1	595	Homo sapiens	Luciferase reporter assay//qRT-PCR//Western blot	Functional MTI	20655737
MIRT000701	hsa-miR-193b-3p	Homo sapiens	ESR1	2099	Homo sapiens	Luciferase reporter assay//Microarray	Functional MTI	19684618
MIRT004413	hsa-miR-193b-3p	Homo sapiens	PLAU	5328	Homo sapiens	Western blot//Luciferase reporter assay	Functional MTI	19701247
MIRT004414	hsa-miR-193b-3p	Homo sapiens	PRAP1	118471	Homo sapiens	Luciferase reporter assay	Functional MTI	21779487
MIRT004663	hsa-miR-193b-3p	Homo sapiens	MCL1	4170	Homo sapiens	Luciferase reporter assay//Western blot	Functional MTI	20103677
MIRT005516	hsa-miR-193b-3p	Homo sapiens	ETS1	2113	Homo sapiens	Luciferase reporter assay//qRT-PCR//Western blot	Functional MTI	20655737
MIRT006983	hsa-miR-193b-3p	Homo sapiens	YWHAZ	7534	Homo sapiens	Luciferase reporter assay//Reporter assay;Proteomics	Functional MTI	21512034
MIRT006985	hsa-miR-193b-3p	Homo sapiens	SHMT2	6472	Homo sapiens	Luciferase reporter assay//Reporter assay;Proteomics	Functional MTI	21512034
MIRT006986	hsa-miR-193b-3p	Homo sapiens	AKR1C2	1646	Homo sapiens	Luciferase reporter assay//Reporter assay;Proteomics	Functional MTI	21512034
MIRT000222	hsa-miR-195-5p	Homo sapiens	WEE1	7465	Homo sapiens	ELISA//GFP reporter assay//Luciferase reporter assay//Microarray//qRT-PCR//Western blot	Functional MTI	19823043
MIRT000223	hsa-miR-195-5p	Homo sapiens	E2F3	1871	Homo sapiens	Immunohistochemistry//Luciferase reporter assay//Northern blot//qRT-PCR//Western blot//Reporter assay;Western blot;qRT-PCR;Other	Functional MTI	19441017

MIRT000223	hsa-miR-195-5p	Homo sapiens	E2F3	1871	Homo sapiens	Luciferase reporter assay//Western blot	Functional MTI	22217655
MIRT000224	hsa-miR-195-5p	Homo sapiens	CDK6	1021	Homo sapiens	Reporter assay;Western blot;qRT-PCR;Other	Functional MTI	19441017
MIRT000225	hsa-miR-195-5p	Homo sapiens	CCND1	595	Homo sapiens	Luciferase reporter assay//Western blot	Functional MTI	22217655
MIRT000225	hsa-miR-195-5p	Homo sapiens	CCND1	595	Homo sapiens	Luciferase reporter assay	Functional MTI	23383003
MIRT006246	hsa-miR-195-5p	Homo sapiens	CCND3	896	Homo sapiens	Luciferase reporter assay//Western blot	Functional MTI	22217655
MIRT006245	hsa-miR-195-5p	Homo sapiens	TBCCD1	55171	Homo sapiens	Luciferase reporter assay//Western blot	Functional MTI	22217655
MIRT006252	hsa-miR-195-5p	Homo sapiens	CDK4	1019	Homo sapiens	Luciferase reporter assay	Functional MTI	22289176
MIRT004273	hsa-miR-195-5p	Homo sapiens	VEGFA	7422	Homo sapiens	ELISA//Luciferase reporter assay	Functional MTI	18320040
MIRT004669	hsa-miR-195-5p	Homo sapiens	CCL4	6351	Homo sapiens	ELISA//Immunoprecipitaion//qRT-PCR//Western blot	Functional MTI	20952681
MIRT004937	hsa-miR-195-5p	Homo sapiens	KRT7	3855	Homo sapiens	Immunoblot//qRT-PCR	Non-Functional MTI (Weak)	19378336
MIRT005362	hsa-miR-195-5p	Homo sapiens	BCL2	596	Homo sapiens	Luciferase reporter assay//qRT-PCR//Western blot	Functional MTI	20727858
MIRT006235	hsa-miR-195-5p	Homo sapiens	SLC2A3	6515	Homo sapiens	Luciferase reporter assay//Western blot	Functional MTI	22265971
MIRT006995	hsa-miR-195-5p	Homo sapiens	CDC42	998	Homo sapiens	Luciferase reporter assay	Functional MTI	22802111
MIRT007054	hsa-miR-195-5p	Homo sapiens	CAB39	51719	Homo sapiens	Immunocytochemistry//In situ hybridization//Northern blot//qRT-PCR//Western blot	Functional MTI	22844503
MIRT007169	hsa-miR-195-5p	Homo sapiens	CHUK	1147	Homo sapiens	Luciferase reporter assay	Functional MTI	23487264
MIRT007170	hsa-miR-195-5p	Homo sapiens	TAB3	257397	Homo sapiens	Luciferase reporter assay	Functional MTI	23487264
MIRT007219	hsa-miR-195-5p	Homo sapiens	MBD1	4152	Homo sapiens	Luciferase reporter assay	Functional MTI	23349673
MIRT007237	hsa-miR-195-5p	Homo sapiens	CCNE1	898	Homo sapiens	Luciferase reporter assay	Functional MTI	23383003
MIRT007370	hsa-miR-195-5p	Homo sapiens	BCL2L2	599	Homo sapiens	Luciferase reporter assay//Western blot	Functional MTI	23526568
MIRT000018	hsa-miR-222-3p	Homo sapiens	STAT5A	6776	Homo sapiens	qRT-PCR//Luciferase reporter assay//Western blot	Functional MTI	20489169
MIRT000131	hsa-miR-222-3p	Homo sapiens	CDKN1B	1027	Homo sapiens	Luciferase reporter assay//Western blot//Western blot;Other//Northern blot	Functional MTI	17569667
MIRT000131	hsa-miR-222-3p	Homo sapiens	CDKN1B	1027	Homo sapiens	qRT-PCR//Luciferase reporter assay//Western blot	Functional MTI	19153141
MIRT000131	hsa-miR-222-3p	Homo sapiens	CDKN1B	1027	Homo sapiens	Western blot//Northern blot	Functional MTI	19107213
MIRT000131	hsa-miR-222-3p	Homo sapiens	CDKN1B	1027	Homo sapiens	Luciferase reporter assay//Western blot	Functional MTI	19859555

MIRT000131	hsa-miR-222-3p	Homo sapiens	CDKN 1B	1027	Homo sapiens	Luciferase reporter assay//qRT-PCR//Western blot	Functional MTI	18417445
MIRT000131	hsa-miR-222-3p	Homo sapiens	CDKN 1B	1027	Homo sapiens	Luciferase reporter assay	Functional MTI	19150885
MIRT000131	hsa-miR-222-3p	Homo sapiens	CDKN 1B	1027	Homo sapiens	Luciferase reporter assay	Functional MTI	18983236
MIRT000131	hsa-miR-222-3p	Homo sapiens	CDKN 1B	1027	Homo sapiens	Luciferase reporter assay	Functional MTI	18246122
MIRT000131	hsa-miR-222-3p	Homo sapiens	CDKN 1B	1027	Homo sapiens	Northern blot//qRT-PCR//Western blot	Functional MTI	20018759
MIRT000131	hsa-miR-222-3p	Homo sapiens	CDKN 1B	1027	Homo sapiens	Immunohistochemistry//In situ hybridization//Luciferase reporter assay//Northern blot//Western blot//Reporter assay;Western blot	Functional MTI	19424584
MIRT000131	hsa-miR-222-3p	Homo sapiens	CDKN 1B	1027	Homo sapiens	Western blot//Reporter assay	Functional MTI	17721077
MIRT000131	hsa-miR-222-3p	Homo sapiens	CDKN 1B	1027	Homo sapiens	Luciferase reporter assay	Functional MTI	17627278
MIRT000131	hsa-miR-222-3p	Homo sapiens	CDKN 1B	1027	Homo sapiens	qRT-PCR//Western blot	Functional MTI	18708351
MIRT000131	hsa-miR-222-3p	Homo sapiens	CDKN 1B	1027	Homo sapiens	Reporter assay	Functional MTI	17914108
MIRT000131	hsa-miR-222-3p	Homo sapiens	CDKN 1B	1027	Homo sapiens	Reporter assay	Functional MTI	18413744
MIRT000135	hsa-miR-222-3p	Homo sapiens	SOD2	6648	Homo sapiens	Flow//Luciferase reporter assay//Microarray//qRT-PCR//Western blot	Functional MTI	19487542
MIRT000136	hsa-miR-222-3p	Homo sapiens	MMP1	4312	Homo sapiens	Flow//Luciferase reporter assay//Microarray//qRT-PCR//Western blot	Functional MTI	19487542
MIRT000433	hsa-miR-222-3p	Homo sapiens	FOXO 3	2309	Homo sapiens	qRT-PCR//ChIP//Luciferase reporter assay//Western blot//Northern blot	Functional MTI	20388878
MIRT006377	hsa-miR-222-3p	Homo sapiens	DICER 1	23405	Homo sapiens	Luciferase reporter assay	Functional MTI	21761362
MIRT000719	hsa-miR-222-3p	Homo sapiens	CDKN 1C	1028	Homo sapiens	qRT-PCR//Luciferase reporter assay//Western blot	Functional MTI	19153141
MIRT000719	hsa-miR-222-3p	Homo sapiens	CDKN 1C	1028	Homo sapiens	Luciferase reporter assay//Western blot	Functional MTI	19589872
MIRT000719	hsa-miR-222-3p	Homo sapiens	CDKN 1C	1028	Homo sapiens	Luciferase reporter assay//Reporter assay;Other	Functional MTI	18413744
MIRT001779	hsa-miR-222-3p	Homo sapiens	KIT	3815	Homo sapiens	qRT-PCR//Western blot	Functional MTI	18417445
MIRT001779	hsa-miR-222-3p	Homo sapiens	KIT	3815	Homo sapiens	Luciferase reporter assay	Functional MTI	18246122
MIRT001779	hsa-miR-222-3p	Homo sapiens	KIT	3815	Homo sapiens	Luciferase reporter assay	Functional MTI	18983236
MIRT001779	hsa-miR-222-3p	Homo sapiens	KIT	3815	Homo sapiens	Northern blot//qRT-PCR//Western blot	Functional MTI	16365291
MIRT001779	hsa-miR-222-3p	Homo sapiens	KIT	3815	Homo sapiens	Reporter assay	Functional MTI	16330772
MIRT002334	hsa-miR-	Homo	TMED	51014	Homo	Western blot	Functional MTI	21226887

	222-3p	sapiens	7		sapiens			
MIRT002334	hsa-miR-222-3p	Homo sapiens	TMED 7	51014	Homo sapiens	Western blot;qRT-PCR	Functional MTI	20018759
MIRT006064	hsa-miR-222-3p	Homo sapiens	ETS1	2113	Homo sapiens	Luciferase reporter assay//qRT-PCR	Functional MTI	23522449
MIRT003191	hsa-miR-222-3p	Homo sapiens	PPP2R 2A	5520	Homo sapiens	Luciferase reporter assay//Western blot	Functional MTI	20103675
MIRT003191	hsa-miR-222-3p	Homo sapiens	PPP2R 2A	5520	Homo sapiens	Reporter assay;Western blot	Functional MTI	21656127
MIRT003451	hsa-miR-222-3p	Homo sapiens	TIMP3	7078	Homo sapiens	Flow//Immunohistochemistry//Luciferase reporter assay//qRT-PCR//Western blot	Functional MTI	19962668
MIRT003451	hsa-miR-222-3p	Homo sapiens	TIMP3	7078	Homo sapiens	Immunohistochemistry//In situ hybridization//qRT-PCR//Western blot	Functional MTI	22009755
MIRT003451	hsa-miR-222-3p	Homo sapiens	TIMP3	7078	Homo sapiens	ELISA//Luciferase reporter assay//qRT-PCR//Western blot	Functional MTI	22681957
MIRT003756	hsa-miR-222-3p	Homo sapiens	TNFSF 10	8743	Homo sapiens	Western blot	Functional MTI	18246122
MIRT004485	hsa-miR-222-3p	Homo sapiens	FOS	2353	Homo sapiens	qRT-PCR//Luciferase reporter assay//Western blot//Northern blot	Functional MTI	20299489
MIRT004485	hsa-miR-222-3p	Homo sapiens	FOS	2353	Homo sapiens	Luciferase reporter assay	Functional MTI	23400877
MIRT005321	hsa-miR-222-3p	Homo sapiens	ESR1	2099	Homo sapiens	Luciferase reporter assay//qRT-PCR//Western blot	Functional MTI	18790736
MIRT005369	hsa-miR-222-3p	Homo sapiens	BBC3	27113	Homo sapiens	Immunohistochemistry//In situ hybridization//Luciferase reporter assay//Northern blot//Western blot	Functional MTI	20813046
MIRT005586	hsa-miR-222-3p	Homo sapiens	PTEN	5728	Homo sapiens	FACS//Flow//Luciferase reporter assay//Northern blot//Western blot	Functional MTI	20618998
MIRT005586	hsa-miR-222-3p	Homo sapiens	PTEN	5728	Homo sapiens	Flow//Immunohistochemistry//Luciferase reporter assay//qRT-PCR//Western blot	Functional MTI	19962668
MIRT005586	hsa-miR-222-3p	Homo sapiens	PTEN	5728	Homo sapiens	Western blot	Functional MTI	23028614
MIRT005791	hsa-miR-222-3p	Homo sapiens	CORO 1A	11151	Homo sapiens	Western blot	Functional MTI	21226887
MIRT005792	hsa-miR-222-3p	Homo sapiens	TCEA L1	9338	Homo sapiens	Western blot	Functional MTI	21226887
MIRT006728	hsa-miR-222-3p	Homo sapiens	RECK	8434	Homo sapiens	Luciferase reporter assay	Functional MTI	22321642
MIRT006918	hsa-miR-222-3p	Homo sapiens	CERS2	29956	Homo sapiens	Luciferase reporter assay//qRT-PCR//Western blot	Functional MTI	22393241
MIRT000109	hsa-miR-26a-5p	Homo sapiens	HMGA 2	8091	Homo sapiens	Luciferase reporter assay	Functional MTI	17563749
MIRT000110	hsa-miR-26a-5p	Homo sapiens	HMGA 1	3159	Homo sapiens	Luciferase reporter assay	Functional MTI	17563749
MIRT000110	hsa-miR-26a-5p	Homo sapiens	HMGA 1	3159	Homo sapiens	Luciferase reporter assay//Western blot	Functional MTI	22245693
MIRT000111	hsa-miR-26a-5p	Homo sapiens	CCNE 2	9134	Homo sapiens	Luciferase reporter assay//Western blot	Functional MTI	19524505

MIRT000112	hsa-miR-26a-5p	Homo sapiens	CCND2	894	Homo sapiens	Luciferase reporter assay//Western blot	Functional MTI	19524505
MIRT006389	hsa-miR-26a-5p	Homo sapiens	ESR1	2099	Homo sapiens	Luciferase reporter assay	Functional MTI	21610700
MIRT006306	hsa-miR-26a-5p	Homo sapiens	CDK6	1021	Homo sapiens	Luciferase reporter assay//Western blot	Functional MTI	22210897
MIRT001039	hsa-miR-26a-5p	Homo sapiens	LIF	3976	Homo sapiens	ELISA	Non-Functional MTI (Weak)	19011087
MIRT001095	hsa-miR-26a-5p	Homo sapiens	PTEN	5728	Homo sapiens	Western blot//Luciferase reporter assay	Functional MTI	19487573
MIRT001095	hsa-miR-26a-5p	Homo sapiens	PTEN	5728	Homo sapiens	Luciferase reporter assay//Western blot	Functional MTI	20216554
MIRT001095	hsa-miR-26a-5p	Homo sapiens	PTEN	5728	Homo sapiens	GFP reporter assay//Luciferase reporter assay//Western blot	Functional MTI	20080666
MIRT001771	hsa-miR-26a-5p	Homo sapiens	EZH2	2146	Homo sapiens	Luciferase reporter assay//Western blot	Functional MTI	18713946
MIRT001771	hsa-miR-26a-5p	Homo sapiens	EZH2	2146	Homo sapiens	Luciferase reporter assay//qRT-PCR//Western blot	Functional MTI	20478051
MIRT001771	hsa-miR-26a-5p	Homo sapiens	EZH2	2146	Homo sapiens	Immunohistochemistry//qRT-PCR//Western blot	Functional MTI	21199804
MIRT001771	hsa-miR-26a-5p	Homo sapiens	EZH2	2146	Homo sapiens	Immunohistochemistry//Luciferase reporter assay//qRT-PCR//Western blot	Functional MTI	20952513
MIRT001771	hsa-miR-26a-5p	Homo sapiens	EZH2	2146	Homo sapiens	Reporter assay	Functional MTI	18281287
MIRT001772	hsa-miR-26a-5p	Homo sapiens	PLAG1	5324	Homo sapiens	Luciferase reporter assay//Microarray//Reporter assay;Other	Functional MTI	16461460
MIRT002315	hsa-miR-26a-5p	Homo sapiens	SERBP1	26135	Homo sapiens	Immunoprecipitaion//Luciferase reporter assay//Western blot	Functional MTI	17637574
MIRT002340	hsa-miR-26a-5p	Homo sapiens	SMAD1	4086	Homo sapiens	Western blot	Functional MTI	20857419
MIRT002340	hsa-miR-26a-5p	Homo sapiens	SMAD1	4086	Homo sapiens	RNase mapping analysis//Western blot//Reporter assay	Functional MTI	17922609
MIRT002340	hsa-miR-26a-5p	Homo sapiens	SMAD1	4086	Homo sapiens	Luciferase reporter assay	Functional MTI	14697198
MIRT002340	hsa-miR-26a-5p	Homo sapiens	SMAD1	4086	Homo sapiens	Luciferase reporter assay//Western blot//Reporter assay	Functional MTI	18197755
MIRT002340	hsa-miR-26a-5p	Homo sapiens	SMAD1	4086	Homo sapiens	Luciferase reporter assay	Functional MTI	23028144
MIRT003213	hsa-miR-26a-5p	Homo sapiens	RB1	5925	Homo sapiens	GFP reporter assay//Luciferase reporter assay//Western blot	Functional MTI	20080666
MIRT003213	hsa-miR-26a-5p	Homo sapiens	RB1	5925	Homo sapiens	Luciferase reporter assay//Microarray//qRT-PCR//Western blot	Functional MTI	23108995
MIRT003212	hsa-miR-26a-5p	Homo sapiens	MAP3K2	10746	Homo sapiens	GFP reporter assay//Luciferase reporter assay//Western blot	Functional MTI	20080666
MIRT006309	hsa-miR-26a-5p	Homo sapiens	CCNE1	898	Homo sapiens	Luciferase reporter assay//Western blot	Functional MTI	22210897
MIRT003803	hsa-miR-26a-5p	Homo sapiens	SMAD4	4089	Homo sapiens	Western blot	Functional MTI	20857419

MIRT003803	hsa-miR-26a-5p	Homo sapiens	SMAD4	4089	Homo sapiens	Luciferase reporter assay	Functional MTI	23028144
MIRT004615	hsa-miR-26a-5p	Homo sapiens	IFNB1	3456	Homo sapiens	ELISA//Luciferase reporter assay//qRT-PCR	Functional MTI	20130213
MIRT004676	hsa-miR-26a-5p	Homo sapiens	GSK3B	2932	Homo sapiens	Luciferase reporter assay//Microarray//qRT-PCR//Western blot	Functional MTI	20525681
MIRT005587	hsa-miR-26a-5p	Homo sapiens	CPEB2	132864	Homo sapiens	Luciferase reporter assay//Northern blot//qRT-PCR	Functional MTI	20660482
MIRT005588	hsa-miR-26a-5p	Homo sapiens	CPEB3	22849	Homo sapiens	Luciferase reporter assay//Northern blot//qRT-PCR	Functional MTI	20660482
MIRT005589	hsa-miR-26a-5p	Homo sapiens	CPEB4	80315	Homo sapiens	Luciferase reporter assay//Northern blot//qRT-PCR	Functional MTI	20660482
MIRT005751	hsa-miR-26a-5p	Homo sapiens	GDAP1	54332	Homo sapiens	Immunofluorescence//In situ hybridization//Luciferase reporter assay//Northern blot//qRT-PCR//Western blot	Functional MTI	20827281
MIRT005920	hsa-miR-26a-5p	Homo sapiens	MTDH	92140	Homo sapiens	Immunohistochemistry//Luciferase reporter assay//qRT-PCR//Western blot	Functional MTI	20952513
MIRT006711	hsa-miR-26a-5p	Homo sapiens	ABCA1	19	Homo sapiens	Immunoblot//Luciferase reporter assay//qRT-PCR	Functional MTI	22673513
MIRT006712	hsa-miR-26a-5p	Homo sapiens	ARL4C	10123	Homo sapiens	Immunoblot//Luciferase reporter assay//qRT-PCR	Functional MTI	22673513
MIRT007347	hsa-miR-26a-5p	Homo sapiens	NOS2	4843	Homo sapiens	qRT-PCR//Western blot	Functional MTI	23338972
MIRT007374	hsa-miR-26a-5p	Homo sapiens	IL6	3569	Homo sapiens	Luciferase reporter assay	Functional MTI	23389848
MIRT052649	hsa-miR-26a-5p	Homo sapiens	MCL1	4170	Homo sapiens	Western blot	Functional MTI	23750239
MIRT005458	hsa-miR-340-5p	Homo sapiens	MET	4233	Homo sapiens	Immunohistochemistry//Luciferase reporter assay//qRT-PCR//Western blot	Functional MTI	21225860
MIRT006217	hsa-miR-483-5p	Homo sapiens	MAPK3	5595	Homo sapiens	Luciferase reporter assay//Microarray//qRT-PCR//Western blot	Functional MTI	22465663
MIRT004118	hsa-miR-483-3p	Homo sapiens	SMAD4	4089	Homo sapiens	Luciferase reporter assay//qRT-PCR//Western blot	Functional MTI	21112326
MIRT004500	hsa-miR-483-3p	Homo sapiens	BBC3	27113	Homo sapiens	qRT-PCR//Luciferase reporter assay//Western blot//Northern blot	Functional MTI	20388800
MIRT006686	hsa-miR-483-3p	Homo sapiens	PARD3	56288	Homo sapiens	qRT-PCR//Western blot	Functional MTI	22101077
MIRT005774	hsa-miR-616-3p	Homo sapiens	TFPI2	7980	Homo sapiens	In situ hybridization//Luciferase reporter assay//Microarray//qRT-PCR	Functional MTI	21224345
MIRT007325	hsa-miR-616-3p	Homo sapiens	PON1	5444	Homo sapiens	Luciferase reporter assay	Functional MTI	23497787
MIRT000448	hsa-miR-650	Homo sapiens	ING4	51147	Homo sapiens	Luciferase reporter assay//qRT-PCR//Western blot	Functional MTI	20381459
